# Limitations of current machine learning models in predicting enzymatic functions for uncharacterized proteins

**DOI:** 10.1093/g3journal/jkaf169

**Published:** 2025-07-24

**Authors:** Valérie de Crécy-Lagard, Raquel Dias, Nick Sexson, Iddo Friedberg, Yifeng Yuan, Manal A Swairjo

**Affiliations:** Department of Microbiology and Cell Science, University of Florida, Gainesville, FL 32611, United States; Genetic Institute, University of Florida, Gainesville, FL 32611, United States; Department of Microbiology and Cell Science, University of Florida, Gainesville, FL 32611, United States; Department of Microbiology and Cell Science, University of Florida, Gainesville, FL 32611, United States; Department of Veterinary Microbiology and Preventive Medicine, Iowa State University, Ames, IA 50011, United States; Department of Microbiology and Cell Science, University of Florida, Gainesville, FL 32611, United States; Department of Chemistry and Biochemistry, San Diego State University, San Diego, CA 92182, United States; The Viral Information Institute, San Diego State University, San Diego, CA 92182, United States

**Keywords:** paralogs, orthologs, YciO, protein language models, Enzyme Commission, protein family, functional annotation

## Abstract

Thirty to seventy percent of proteins in any given genome have no assigned function and have been labeled as the protein “unknome.” This large knowledge shortfall is one of the final frontiers of biology. Machine learning (ML) approaches are enticing, with early successes demonstrating the ability to propagate functional knowledge from experimentally characterized proteins. An open question is the ability of ML approaches to predict enzymatic functions unseen in the training sets. By integrating literature and a combination of bioinformatic approaches, we evaluated individually Enzyme Commission number predictions for over 450 *Escherichia coli* unknowns made using state-of-the-art ML approaches. We found that current ML methods not only mostly fail to make novel predictions but also make basic logic errors in their predictions that human annotators avoid by leveraging the available knowledge base. This underscores the need to include assessments of prediction uncertainty in model output and to test for “hallucinations” (logic failures) as a part of model evaluation. Explainable artificial intelligence analysis can be used to identify indicators of prediction errors, potentially identifying the most relevant data to include in the next generation of computational models.

## Teaser

Many proteins in any genome, ranging from 30% to 70% of the genome, lack an assigned function. This knowledge gap limits the full use of the vast available genomic data. Machine learning has shown promise in transferring functional knowledge within isofunctional families, but it largely fails to predict novel functions not seen in its training data. Understanding these failures can guide the development of better machine-learning methods to help experts make accurate functional predictions for uncharacterized proteins.

## Introduction

Determining protein function is not an easy task, and 30 yr after the first bacterial genome was sequenced, the functional annotation status of the proteome of most species is far from being accurate or complete, even for model organisms (Ghatak et al. 2019; Wood et al. 2019; de Crécy-lagard et al. 2022; Rocha et al. 2023). Experimental validation of protein function is a painstaking process, and with the explosion of whole genome sequences (Kyrpides 1999; Peterson et al. 2025; Torres et al. 2025), the gap between experimentally validated functions and those predicted through computational methods continues to widen. In UniProtKB (Bateman et al. 2023), the most widely used protein function database (Ramola et al. 2022), the estimates are that less than 0.5% to 15% of proteins have been linked to experimental data (Škunca et al. 2017).

**Fig. 1.**
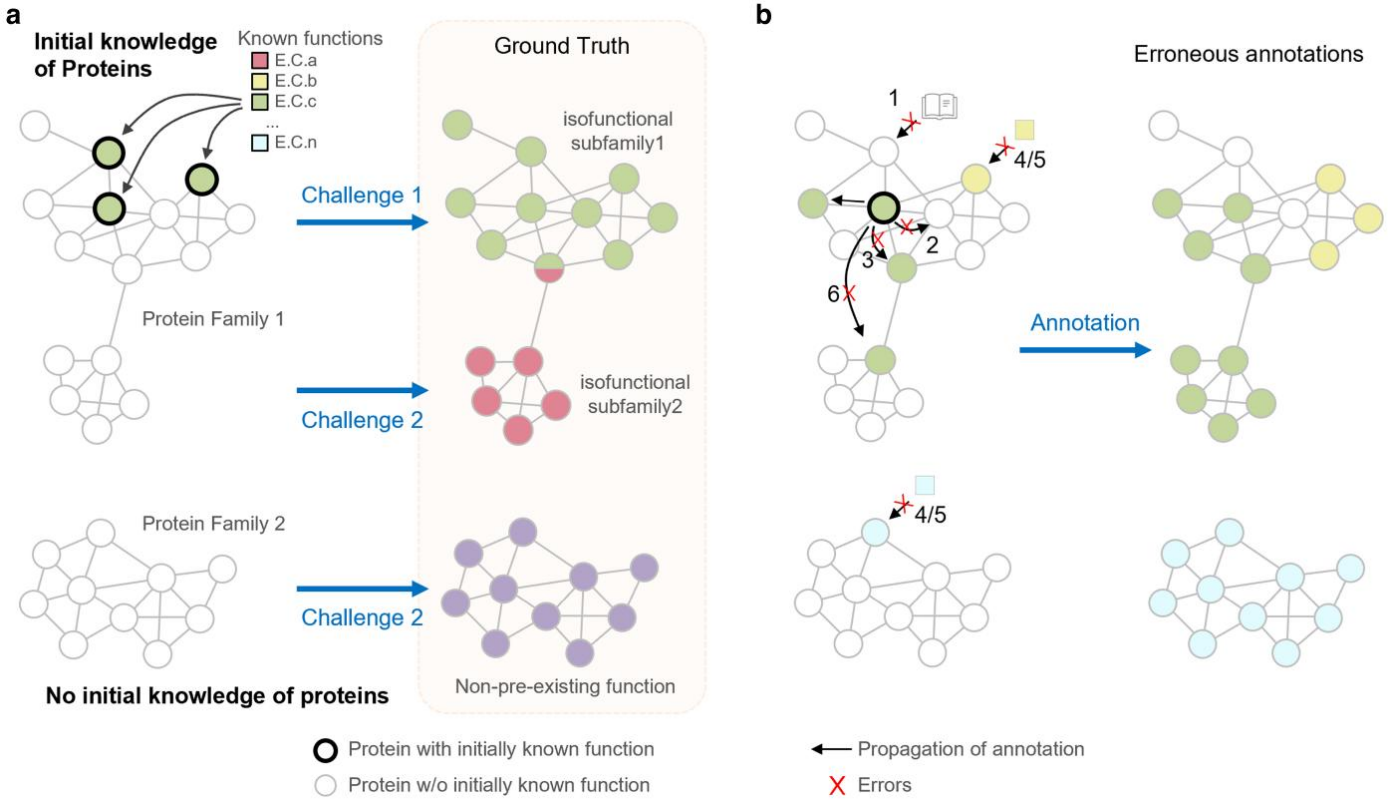
The major 2 types of challenges in accurate protein functional annotation. a) Challenge 1: to propagate the existing knowledge to the correct set of unannotated proteins. Challenge 2: to annotate proteins in a family with none of the members linked to any initial known information. Each circle represents a protein in a family with (bold border) or without (thin border) initially known function. The edges that connect 2 circles represent protein similarity above a preset threshold. The squares represent known functions with E.C.a to E.C.n as examples. b) The errors during propagation of annotation that could lead to erroneous results. The numbering of errors (red crosses) corresponds to the error types described in Table 1.

The process of functional annotation of protein entries in databases starts with capturing information in the literature by biocurators (International Society for Biocuration 2018). This process links experimental characterizations of specific proteins in specific organisms to controlled vocabularies that describe validated functions, such as the Gene Ontology (GO) (Gene Ontology Consortium et al. 2023), the IUPAC Enzyme Commission (EC) numbers (https://iubmb.qmul.ac.uk/enzyme/), or biochemical reaction descriptors (e.g. Rhea (Bansal et al. 2022)). Text mining tools have accelerated the flow of information captured (Soldatos et al. 2015; Poux et al. 2017; Wei et al. 2019). However, this step remains a major bottleneck in the annotation workflow, which can result in mislabeling proteins as “unknown” when a function has been reported in the literature (see type 1 error in Table 1 and Fig. 1).

**Table 1.** Main types of errors that lead to erroneous functional annotations of proteins.

Error types		
False unknowns	Annotated as unknown or general but function is known and published	
	Details	Example
1 Failure to capture the literature	Not captured in any database or captured in some databases but not others Also annotated as vague when precise annotation is known	CT_611 is captured as folylpolyglutamate synthase in KEGG (ctr:CT_611), but this annotation is still not in UniProt as of April 2024 (O84617). See also examples in Price and Arkin (2024)
2 Naming issues	Inconsistent naming of the same entities	See GroEL in Lockwood et al. (2019)
Propagation failures	Captured for one member of a family but not propagated to others	The MptE protein that encodes 6-hydroxymethyl-7,8-dihydropterin pyrophosphokinase in most Archaea (de Crécy-Lagard et al. 2012) is captured in BioCyc for *Methanocaldococcus jannaschii DSM 2661* (https://biocyc.org/gene?orgid=MJ&id=MJ_RS08700-MONOMER) but not propagated to any archaeal homolog
Fusion and multidomain proteins	Only one out of multiple functions is captured or domain shuffling leads to miscalling	See examples in Henryt e al. (2016) and Hegyi and Gerstein (2001)

False knowns	Annotated as precise but wrong (should be general or another annotation) or incomplete	
	Details	Example
3 Multiple functions	Protein has multiple functions because of fusions, moonlighting, or promiscuity, and only one of the functions is captured	For example, A5I019 has 2 functions QueD and PTPS-III, and only one is called in UniProt (de Crécy-Lagard 2014). See also examples in Price and Arkin (2024)
4 Curation mistake	(1) The data were incorrectly captured by a biocurator; (2) functional annotations may become outdated	Ureidoglycolate lyase (Percudani et al. 2013)
5 Experimental mistake	Finding has been refuted by other studies: (1) the published data are inconclusive; (2) inconsistency occurs when different databases or resources provide conflicting functional annotations for the same protein	DUF34 family was annotated as GTP cyclohydrolase 1B (Reed et al. 2021)
6 Overannotation of paralogs	Annotation wrongly propagated to nonisofunctional paralogous groups	See examples in Schnoes et al. (2009), Zallot et al. (2016), and (Rembeza and Engqvist 2021)

Error types 1–6 correspond to the errors numbered in Fig. 1. KEGG: Kyoto Encyclopedia of Genes and Genomes.

Using the principle of sequence similarity, also known as homology transfer, putative functions are assigned to proteins in newly sequenced genomes as a part of the genome annotation process (Seemann 2014; Thibaud-Nissen et al. 2016; Olson et al. 2023). Indeed, most functions of proteins in UniProt have been inferred computationally based on sequence similarity (Bateman et al. 2023). The methods used over the past 30 yr to automatically propagate functional annotations have been extensively reviewed (Storm and Sonnhammer 2002; Friedberg 2006; Lee et al. 2007; Satish Kumar et al. 2007; Devoid et al. 2013). This seemingly simple task can become quite difficult (challenge 1 in Fig. 1), since relying on sequence similarity alone can result in significant annotation errors (Schnoes et al. 2009; Rembeza and Engqvist 2021). These errors can arise from various sources, including human annotation mistakes or intrinsic features of the protein family itself, such as domain shuffling or domain fusions (Table 1). One prevalent error type affecting protein families (Rembeza and Engqvist 2021) is in the misannotation of paralogs (Schnoes et al. 2009; Zallot et al. 2016; Rembeza and Engqvist 2021) caused by the inherent functional complexity of enzyme families (Glasner et al. 2006; Gerlt et al. 2012) (see type 6 error in Table 1 and Fig. 1). Functional diversification through duplication and divergence (Todd et al. 2001; Das et al. 2015; Bordin et al. 2021) results in proteins with high degrees of similarity having different functional roles, creating nonisofunctional paralogous groups. Indeed, even within a single protein family, a difference in a few amino acids can affect substrate binding and/or catalysis, effectively changing the function (Roy et al. 2009; Ribeiro et al. 2020; Precord et al. 2023). Most current genome annotation pipelines annotate paralogs without considering the potential for functional divergence, leading to incorrect annotations for up to 80% of family members, with these types of errors increasing over time (Schnoes et al. 2009; Rembeza and Engqvist 2021).

**Fig. 2.**
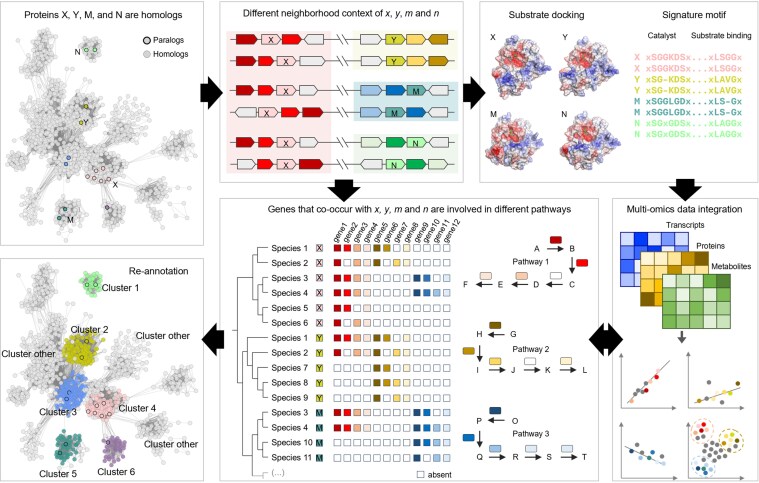
Computational workflow used to separate nonisofunctional paralogs in a protein superfamily. SSNs of protein families can separate paralogs by integrating different types of information, including genomic neighborhood context, structure, cooccurring genes, multiomics data, etc. Arrows indicate the flow of information. The final network shows the separation and reannotation of paralogous subclusters.

Methods for inferring functions that incorporate additional information such as phylogeny, active site analyses, metabolic reconstruction, and sequence similarity networks (SSNs) combined with gene neighborhood or coexpression information can better separate nonisofunctional subfamilies (Zallot et al. 2016, 2021; Ribeiro et al. 2023) (Fig. 2). When hidden Markov models (HMMs) or signature motifs are generated to annotate the subfamilies, they can be integrated into annotation pipelines such as RefSeq (Li et al. 2021) or incorporated into rules such as the Unified Rule used by UniProtKB (MacDougall et al. 2020) additional curation, which has not kept pace with the exponential increase in sequenced genomes. In addition to misassignments among paralogs increasing with evolutionary distance, distinguishing between sub- and neofunctionalization can be problematic (Birchler 2025).

The task of functional annotation becomes even more challenging when one focuses on proteins that have not previously been functionally characterized. Estimates suggest that 30% to 70% of proteins in any given genome are in this “unknown” category (Ghatak et al. 2019; Wood et al. 2019; Lobb et al. 2020; Sajid et al. 2024). Some of these “unknowns” are likely to fill functional roles not yet linked to a specific gene (Karp 2004; Lespinet and Labedan 2006; Chen and Vitkup 2007; Niehaus et al. 2015). Others are likely to be nonorthologous replacements (or convergent evolution), where different (structurally diverse) protein families overlap in functional roles (Ribeiro et al. 2023). Finally, we expect some novel biological functions and predicting these requires extrapolation beyond the current knowledge base.

In summary, the annotation of a new genome faces 2 challenges: (i) to identify genes whose likely function can be inferred by propagating known annotations and (ii) to identify genes with potentially novel functions and make predictions. This is a thorny path with many different types of errors that can be made (Fig. 1b and Table 1). Computational approaches, however innovative, cannot prove a particular protein function; experimental validation is still necessary. Proof of function is not necessarily straightforward, as demonstrating a protein capable of participating in a chemical reaction is not proof that the protein has the responsibility of that function in the cellular context where it is identified (García-Contreras et al. 2012 ; Copley 2015). For proteins with available experimental evidence of function in 1 context, the computational propagation of the assigned functional role is the logical next step (Lee et al. 2007), although as described above, similarity does not guarantee functional conservation. For unknown proteins, the increasing amounts of genome-wide/transcriptome-wide/metabolome-wide experimental data can be used in combination with structural knowledge to develop predictions that can be tested experimentally (Hanson et al. 2010). Scientists are increasingly successful in these types of endeavors in a range of systems (Dembech et al. 2023; Rodríguez del Río et al. 2024), and there is tremendous hope that artificial intelligence (AI) methods will accelerate the pace at which predictions can be made.

**Fig. 3.**
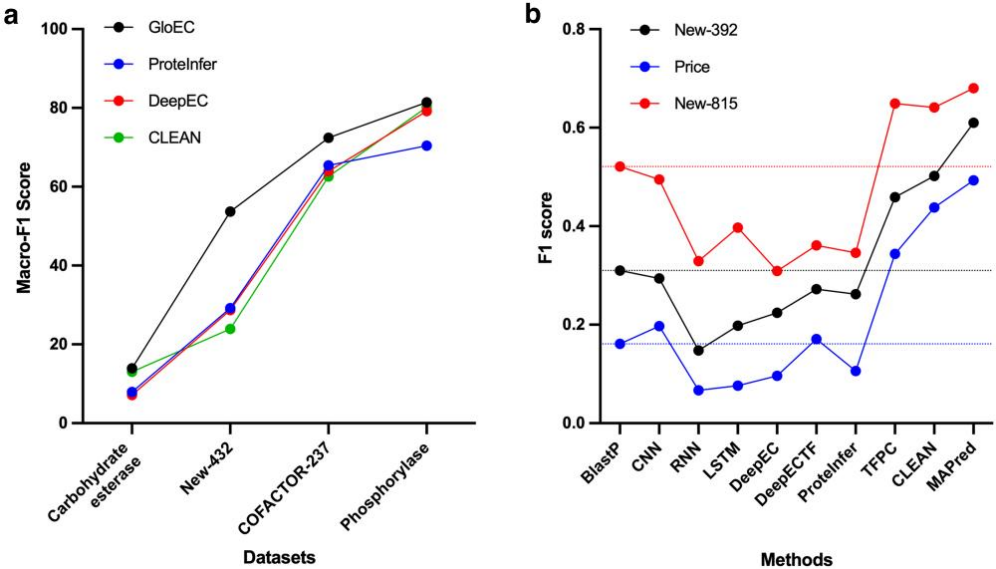
Comparison of combined macro-F1 or regular F1 scores using different ML methods to predict EC numbers. The F1 score is the harmonic mean of precision and recall. Recall is the proportion of true positives correctly identified out of all actual positives, while precision is the proportion of true positives among all predicted positives. A “positive” refers to an instance that actually belongs to the class of interest (here, the correct EC number). The macro-F1 score is calculated by computing the F1 score for each EC number category independently and then averaging these scores across EC numbers. a) Comparison of macro-F1 scores generated using 4 methods (GloEC, Protein Infer, DeepEC, and CLEAN) on 4 datasets (Carbohydrate esterase, New-432, Cofactor-237, and phosphorylase) (data extracted from Huang et al. 2024). b) Comparison of F1 scores generated using 8 methods including BlastP, DeepECTF, DeepEC, and CLEAN on 3 datasets (New-815, New-392, and Price) (data extracted from Rong et al. 2024). Dotted lines mark the BlastP scores. See initial publications for details and references for methods and datasets.

For challenge 1, there has been a recent explosion of publications reporting the use of pretrained protein language models (PLMs) to predict protein functions (Ardern et al. 2023; Ayres et al. 2023; Derry et al. 2025; Durairaj et al. 2023; Kim et al. 2023; Prabakaran and Bromberg 2024; Rodríguez del Río et al. 2024; Hwang et al. 2024). Models have been developed to link protein sequences directly to vocabularies such as GO terms (Radivojac et al. 2013; Jiang et al. 2016; Zhou et al. 2019) or EC (EC classification) numbers (Hamamsy et al. 2023). EC numbers are a set of 4 numbers that are hierarchical with the first number the most general classification and the last number the most specific and whose purpose is to standardize the description of enzymatic activities. PLMs have been reported to accurately predict the first 2 digits of the EC number but not the last 2 (Sanderson et al. 2023). GloEC (Huang et al. 2024) and MAPred (Rong et al. 2024) are 2 recent PLM-based EC prediction tools that achieve precision and recall metrics ranging from 10% to 80%, depending on the test set (Fig. 3). When training data contain well-characterized proteins with well-described structures (e.g. cofactor-237 set) or well-separated functional groups (e.g. phosphorylase set), machine learning (ML) methods are quite proficient (Fig. 3a). Once the structure of the training data is more complex, and the algorithm must distinguish among similar enzymes with different functions, such as the carbohydrate dataset that comprised ∼350 glycosyl hydrolases or if the dataset contains enzymes not included in any training sets such as the Price dataset (Price and Arkin 2024), ML methods do poorly (Fig. 3b).

Several independent studies agree that the number of proteins in the model Gram-negative *Escherichia coli* K12 not linked to precise molecular and/or biological functions is between 1,200 and 1,400, or a quarter of the encoded proteins (Ghatak et al. 2019). Some of these proteins are likely responsible for functions that have been described in *E. coli* but never linked to a gene (called orphan enzymes (Karp 2004; Danchin et al. 2018; Kobras et al. 2021)) or functions that might have been described in another organism but could also be present in *E. coli.* What percentage of these currently unannotated proteins represent novel functions is difficult to determine. A recent study labeled over 450 of the unknowns in the model bacteria *E. coli* with substrate-level specificity ECs (all 4 numbers) using a supervised ML-based DeepECTransformer (DeepECTF) platform and validated 3 of these predictions in vitro (Kim et al. 2023). If corroborated, this report could be a real breakthrough in predictive modeling by linking true unknowns with their function, a process that usually takes years (Hanson et al. 2010; Niehaus et al. 2015). We designed a study to compare human curation to the reported DeepECTF predictions and examined in detail the potential of this new approach.

## Methods

### Bioinformatic analyses and data mining used to evaluate EC number prediction

The UniProt (www.uniprot.org) (Bateman et al. 2023), InterPro (www.ebi.ac.uk/interpro/) (Paysan-Lafosse et al. 2023), NCBI (www.ncbi.nlm.nih.gov) (NCBI Resource Coordinators 2016), BioCyc (www.biocyc.org) (Karp et al. 2019), and KEGG (Kyoto Encyclopedia of Genes and Genomes) (Kanehisa et al. 2021) knowledge bases were used to gather information and evaluate individually the 453 *E. coli* proteins of unknown function annotated with an EC number using the DeepECTF platform (Kim et al. 2023). In addition, PaperBLAST was used to query literature on each entry (https://papers.genomics.lbl.gov/cgi-bin/litSearch.cgi) (Price and Arkin 2017).

### Comparing human curation with DeepECTF predictions

Based on the evidence gathered manually, the DeepECTF predictions for the 453 target unknowns were grouped into different categories with confidence scores (CSs) (Supplementary Table 1). Concordant predictions for all 4 EC numbers were given a CS of 2. These were of 2 types: proteins with the identical functional annotation already present in UniProt (with or without an EC number) and proteins with functions not captured by UniProt. Discordant predictions were cases where (i) another protein was known to perform the same function and experimental data showed that there were no redundancies; (ii) the predicted function was known to be absent in *E. coli*; and (iii) the published literature or comments in UniProt provided evidence for a different function were given a CS of 0. When proteins were members of families with many paralogous subgroups but no literature disproving the prediction was found and in other cases of uncertain calls, a CS of 1 was given.

### Structural and phylogenetic analyses

Crystal structures were retrieved from the Protein Data Bank (www.rcsb.org) (Burley et al. 2023) and visualized using PyMOL (www.pymol.org) (The PyMOL Molecular Graphics System, Version 1.8, Schrödinger, LLC). Structure-based multisequence alignment derived from available crystal structures and AlphaFold structural models (https://alphafold.ebi.ac.uk/) were generated using PROMALS3D (http://prodata.swmed.edu/promals3d/promals3d.php) (Pei et al. 2008) and ESPript (espript.ibcp.fr/ESPript/ESPript). The percent conservation scores were calculated using the program AL2CO (http://prodata.swmed.edu/al2co/al2co.php) (Pei and Grishin 2001 ) using 4264 TsaC/Sua5 sequences and 1955 YciO sequences (Supplementary Data 1 and 2). To generate the TsaC/YciO phylogenetic tree, proteins were aligned using MUSCLE (Edgar 2004). The alignment was trimmed using BMGE (Criscuolo and Gribaldo 2010) and used to build the maximum likelihood tree using FastTree (Price et al. 2010) with LG + CAT model with bootstrap (1,000 replicates) and visualized using iTOL (Letunic and Bork 2021).

### SSNs and gene neighborhood analyses

As an example of the computational analysis that is required to separate paralogs described in Fig. 1, we generated SSNs and the corresponding gene neighborhood networks for the PF01300 using EFI Enzyme Similarity Tool (EFI-EST, efi.igb.illinois.edu/efi-est) (Zallot et al. 2019). Briefly, 54,820 sequences of the PF01300 family between 150 and 350 aa in length were retrieved from UniProt and subjected to EFI-EST. Each node in the network represents 1 or multiple sequences that share no less than 70% identity. The initial SSN was generated with an alignment score (AS) cutoff set such that each connection (edge) represented a sequence identity above 40%. The nodes of paralogs were colored as given in the legend and visualized using Cytoscape (3.10.1) (Shannon et al. 2003). More SSNs were created by gradually increasing the AS cutoff in small increments (usually by 5 AS units). This process was repeated until most clusters were homogeneous in color (AS = 70). The genome neighborhood graphs were generated using Gene Graphics (https://genegraphics.net/) (Harrison et al. 2018).

### Miscellaneous data extraction and analysis

Open AI ChatGPT 4.o was used to extract data from tables in publications and generate figures (https://chatgpt.com/) on August 20 using the prompt: “Extract data from Table X in attached pdf file.”

### Explainable AI methods

To enhance the interpretability of DeepECTF's multilabel enzyme function predictions, we incorporated an explainable AI (XAI) module based on the local interpretable model-agnostic explanation (LIME) framework (Ribeiro et al. 2016). The approach was specifically adapted to address the multilabel nature of EC number predictions and to provide both local and global insights into the protein sequence segments or residues driving model decisions. Traditional LIME is designed for binary or multiclass outputs, but enzyme function prediction often involves multilabel assignments. To address this, we implemented a multilabel adaptation of LIME, which constructs independent explanation pipelines for each possible label. For each protein sequence input, the XAI module isolates the probability output for a single EC label using the model's sigmoid activation, enabling LIME to generate label-specific explanations. To optimize computational efficiency, explanations are generated primarily for the label with the highest predicted probability for each input sequence, focusing interpretability efforts on the most relevant predictions.

To balance explanation quality with computational feasibility, local LIME explanations are computed for a representative subsample of up to 500 protein sequences at a time. For each sequence, the module generates a local feature importance map, highlighting which residues in the protein sequence most influenced the model's prediction for the selected EC label. For proteins outside this subsample, residue-level importance is estimated based on the aggregate statistics from the explained subset. This approach enables the extraction of both local (residue-level) and global (dataset-level) feature importance profiles, which are subsequently exported for downstream analysis. To further dissect model behavior, feature importance scores derived from LIME explanations were stratified by prediction type (correct predictions [CORs], paralog errors, nonparalog errors, and repetitions [REPs]; see Supplementary Table 1). Residue-level importance values are normalized within each error type, and summary plots are generated to visualize the distribution and magnitude of important sequence segments across different error categories.

The XAI module is fully integrated into the DeepECTF workflow, allowing users to generate explainability outputs alongside standard predictions without additional user intervention (https://github.com/Dias-Lab/XAI_DeepProZyme). All explanation results, including local and global feature importance scores, are made available in standard tabular formats for further interpretation or visualization. This explainable AI approach provides actionable insights into the decision-making process of DeepECTF, supporting both the validation of CORs and the systematic investigation of model failures, facilitating the development of more robust and trustworthy protein function prediction systems.

## Results

### Most correctly AI-predicted EC numbers are generic or already in the training set

**Fig. 4.**
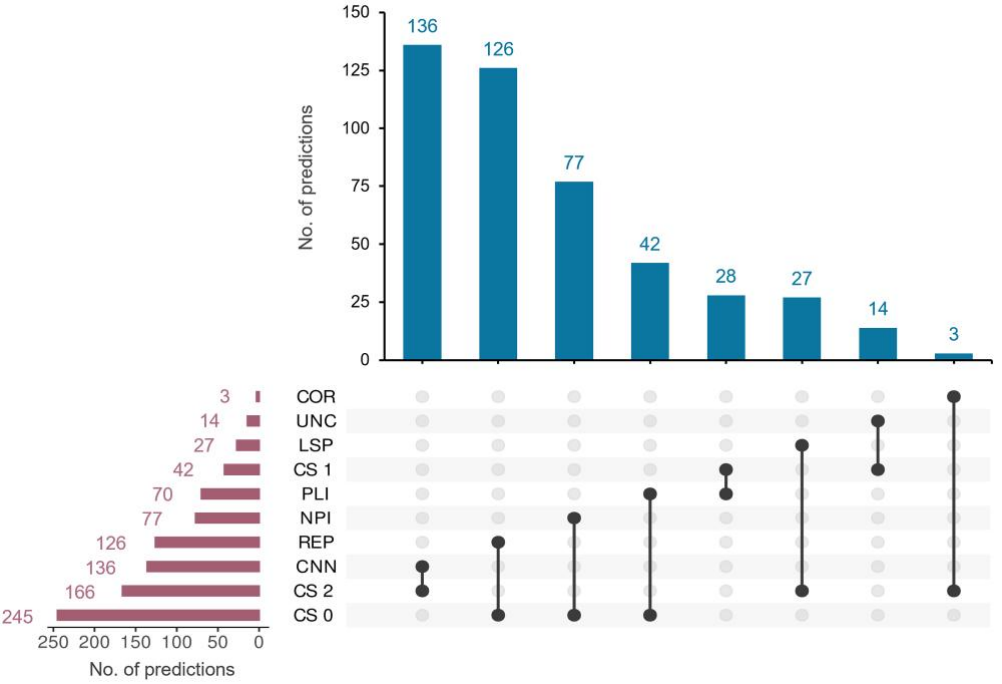
Classification of DeepECTF predictions. The 453 EC number predictions for 453 *E. coli* unknowns were manually classified into categories by comparing the EC number in the UniProt database and labeled with CSs from 0 to 2. Data extracted from Supplementary Table 1. COR, correct prediction; UNC, uncertain; LSP, less precise; PLI, paralogs incorrect; NPI, nonparalog incorrect; REPs, repetitions; CNN, correct but not novel; CS, confidence score.

We analyzed 453 *E. coli* proteins functionally annotated in the Kim et al. (2023) study (Supplementary Table 1). One hundred and twenty-one had the same EC number in the August 2024 corresponding UniProt annotation (Bateman et al. 2023), and 87% of these were in the version used to generate the training dataset (Fig. 4; Supplementary Table 1a) making these an example of training data contamination. Fifteen proteins had the exact same function labeled in UniProt but without an EC number or with a partial EC number (Fig. 4; Supplementary Table 1b and c) and are examples of successful annotation propagation (Fig. 1). These 136 cases were all considered correct but not novel (CNN) predictions with CSs of 2 (Fig. 4).

The remaining 317 predictions can be split into proteins with partial or different EC numbers present in the UniProt annotation (63 cases; Supplementary Table 1b) or with no EC number in the corresponding UniProt entry (254 cases; Supplementary Table 1c). For 27 predictions, more generic annotations than the existing UniProt annotations were given (less precise cases or LSP in Supplementary Table 1b and c). These were also given a CS of 2. The 290 remaining cases were subject to human curation. We combined PaperBLAST searches and UniProt and EcoCyc data analyses to group the 290 DeepECTF predictions into different categories with CSs from 0 to 2 (Supplementary Table 1; Fig. 4). The 42 predictions that could neither be validated nor refuted were labeled as uncertain with a CS of 1. Three cases, including YgfF discussed below, were validated by publications not captured in UniProt and can be considered successful predictions (CSs of 2). Combining the CORrect (COR, 3), the CNN (136), and the LSP or generic (27) predictions brings the number of CORs to 166 (36.6%). Two hundred and forty-five predictions (54%) were inconsistent with the existing evidence and given CSs of 0 (Supplementary Table 1b and c).

### Manual analyses reveal logical inconsistencies in AI predictions of unknowns

The 245 predictions with CSs of 0 can be separated into 2 categories: those with published evidence refuting the annotation (77 of nonparalog incorrect [NPI] and 42 paralog incorrect [PLI] in Supplementary Table 1b and c) and those that were replications of identical EC numbers (126 REPs in Supplementary Table 1b and c). Examples of the first type (*n* = 119) are given in Table 2. For example, YjhQ/b4307 is predicted to be a mycothiol synthase (EC 2.3.1.189), but mycothiol is not a molecule synthesized by *E. coli*, and the remaining pathway genes are absent from the genome (BioCyc ID: PWY1G-0). YrhB/b3446 is predicted to be a 6-carboxytetrahydropterin synthase (EC 4.1.2.50), but *E. coli* already encodes this enzyme (QueD/b2765) and a *queD* mutant lacks this activity (Zallot et al. 2017).

**Table 2.** Example where experimental evidence contradicts the DeepECTF predictions.

Locus tag	UniProt EC	UniProt function	DeepEC EC	DeepEC function	Notes
**Example of overpropagation mistakes**					
b2736	1.1.1.411	L-Threonate dehydrogenase	1.1.1.60	2-Hydroxy-3-oxopropionate reductase	In vivo and in vitro data validate these proteins as involved D-Threonate degradation (Zhang et al. 2016 )
b2738	4.1.1.104	3-Oxo-tetronate 4-phosphate decarboxylase	4.1.2.17	L-Fuculose-phosphate aldolase	
b2739	5.3.1.35	2-Oxo-tetronate isomerase	5.3.1.22	Hydroxypyruvate isomerase	
b4403	2.1.1.-	Uncharacterized tRNA/rRNA methyltransferase LasT	2.1.1.200	tRNA (cytidine32/uridine32-2′-O)-methyltransferase	This EC 2.1.1.200 activity is catalyzed by TrmJ/b2532, and the mutant is devoid of the Um/Cm_32_ modification (Purta et al. 2006)
b2160	2.7.1.-	Uncharacterized sugar kinase YeiI	2.7.1.83	Pseudouridine kinase	This activity is catalyzed by PsuK/b2166, and the mutant cannot use Psi as a C source (Preumont et al. 2008)
b3038	6.3.1.-	Putative acid–amine ligase YgiC	6.3.1.8	Glutathionylspermidine synthase	This activity is catalyzed by Gsp, and YgiC does not have the same activity (Sui et al. 2012)
b1267	None	Uncharacterized protein YciO	2.7.7.87	L-Threonylcarbamoyladenylate synthase	YciO is a paralog of TsaC but does catalyze the same activity (El Yacoubi et al. 2009; Gerdes et al. 2011)
**Example of refuted predictions**					
b2438	None	Bacterial microcompartment shell protein EutK (ethanolamine utilization protein EutK)	2.1.1.223	tRNA1Val (adenine37-N6)-methyltransferase	This activity is catalyzed by TrmN6/b2575, and the mutant does not make the modification (Golovina et al. 2009)
b0254	None	HTH-type transcriptional regulator PerR (peroxide resistance protein PerR)	2.4.2.29	tRNA-guanosine34 preQ_1_ transglycosylase	This activity is catalyzed by Tgt/b0406, and the mutant does not insert preQ_1_ in tRNA (Noguchi et al. 1982)
b3446	None	Uncharacterized protein YrhB	4.1.2.50	6-Carboxytetrahydropterin synthase	This activity is catalyzed by QueD/b2765), and the mutant lacks this activity (Zallot et al. 2017)
b4307	2.3.1.-	Uncharacterized N-acetyltransferase YjhQ	2.3.1.189	Mycothiol synthase	Mycothiol is not synthesized by *E. coli*, and the remaining pathway enzymes are absent (BioCyc ID: PWY1G-0)

Replication of identical EC numbers occurred for 126 proteins (Fig. 4; Supplementary Table 1b and c). REPs of EC numbers do occur in bacterial genomes, particularly with families that, despite having 4 EC numbers, have generic functions. For example, histidine kinases with different substrate specificities are frequent, with 29 annotated in *E. coli* (Supplementary Table 1d, top). However, analysis of the protein family domain membership showed that most of the REPs in these data were errors, except for the less specific annotation cases classified as CORs above (Supplementary Table 1c). For example, out of the 12 proteins annotated as histidine kinase (EC 2.7.13.3) in the 453 DeepECTF predictions, none of them have sequence similarity to histidine kinase families, and 8 have been annotated with different and experimentally validated functions (such as ferric enterobactin transport protein FepE for b0587) (Supplementary Table 1c). For the 15 proteins annotated as “protein-Npi-phosphohistidine-sugar phosphotransferase” (EC 2.7.1.69) (or PTS family proteins), 4 were indeed PTS transporters but were given less specific annotations than the ones in UniProt (Supplementary Table 1c). The 11 remaining were part of transporter families not related to PTS (Supplementary Table 1b). This type of error may be due to inherent limitations in how AI methods operate. Indeed, if the input features in the training data lack the biological structure and cannot leverage information to distinguish between different functions, the model is expected to make frequency-dependent predictions that reflect the training data, as demonstrated with histidine kinases.

### Correct separation of paralogous group can confirm or refute AI-based functional predictions

Many of the incorrect or uncertain predictions (CSs of 0 or 1) were part of protein families with paralogs (Fig. 4; Supplementary Table 1b and c). For example, b2100 was annotated as a dehydro-2-deoxygluconokinase (EC 2.7.1.92) using DeepECTF and as an uncharacterized sugar kinase YegV (EC 2.7.1.-) in UniProt (Supplementary Table 1b). These 2 predictions differ by the fourth or last position of the EC number that specifies substrate specificity. This protein is a member of a superfamily of sugar kinases with multiple nonisofunctional paralogous subgroups that phosphorylate different substrates (Supplementary Table 1d, bottom). The dehydro-2-deoxygluconokinase (EC 2.7.1.92) activity is encoded by another member of this superfamily KdgK/b3526 (Supplementary Table 1d, bottom). Here, DeepECTF predicted correctly the first 3 digits of the EC number but not the last, making an overpropagation mistake (error 6 in Table 1 and Fig. 1). Correctly separating nonisofunctional paralogous subgroups in a superfamily is difficult, requiring extensive examination of all of the evidence and oftentimes experiments.

We performed an additional, in-depth analysis of the 3 proteins described using in vitro assays in the Kim et al. (2023) study (YgfF, YjdM, and YciO) and show that in vitro functionality does not always correspond with in vivo functionality.

#### YgfF analysis

YgfF is a member of the large short-chain dehydrogenase/reductase (SDR) superfamily (IPR002347). The Oppermann and Persson groups developed a nomenclature system and HMM-based classification (http://www.sdr-enzymes.org/) that distinguish different functional subgroups of the SDR superfamily (Persson et al. 2009; Kallberg et al. 2010). This resource predicts YgfF is part of the SDR63C/Glucose 1-dehydrogenase subgroup, the activity predicted and validated in the Kim et al. (2023) study. This prediction demonstrates the accurate propagation of functional annotation and is a successful prediction.

#### YjdM analysis

**Fig. 5.**
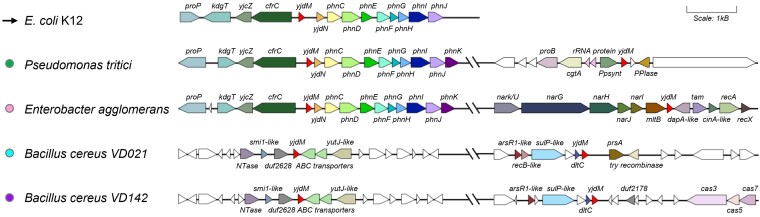
Gene neighborhoods and metabolic reconstructions do not link YjdM to phosphonate degradation. Most *yjdM* genes are not near phosphonate degradation operons. A SSN of 11,986 IPR004624 family members was generated, and the corresponding genomic neighborhood information is available Supplementary Table 4.

YjdM was predicted and shown to catalyze phosphonoacetate hydrolase (PhnA) (EC 3.11.1.2) activity in vitro. In *E. coli*, the *yjdM* gene is located upstream of the methylphosphonate catabolism operon (*phnCDEFGHIJKLMNOP*) (Fig. 5). Underscoring the challenges in annotations, the initial report that the protein was involved in phosphonate catabolism was later refuted with additional genetic analyses (Metcalf and Wanner 1993). The experimentally validated PhnA is part of a nonhomologous family and expression of members of this family in *E. coli* suggested that PhnA activity was not present in this organism (Kulakova et al. 1997). Genome neighborhoods of *phnA* show strong clustering with genes encoding phosphonoacetate transporters, phosphonoacetate sensing regulators, and, in some cases, enzymes involved in 2-aminoethylphosphonate catabolism (Kulakova et al. 2001). However, except for *E. coli*, *yjdM* genes are generally not close to phosphonate catabolism or transport genes (Fig. 5). In conclusion, the PhnA activity observed in vitro is not supported, and additional in vivo experiments are required to confirm the biological role of this enzyme. This prediction was given a CS of 1.

#### YciO analysis

**Fig. 6.**
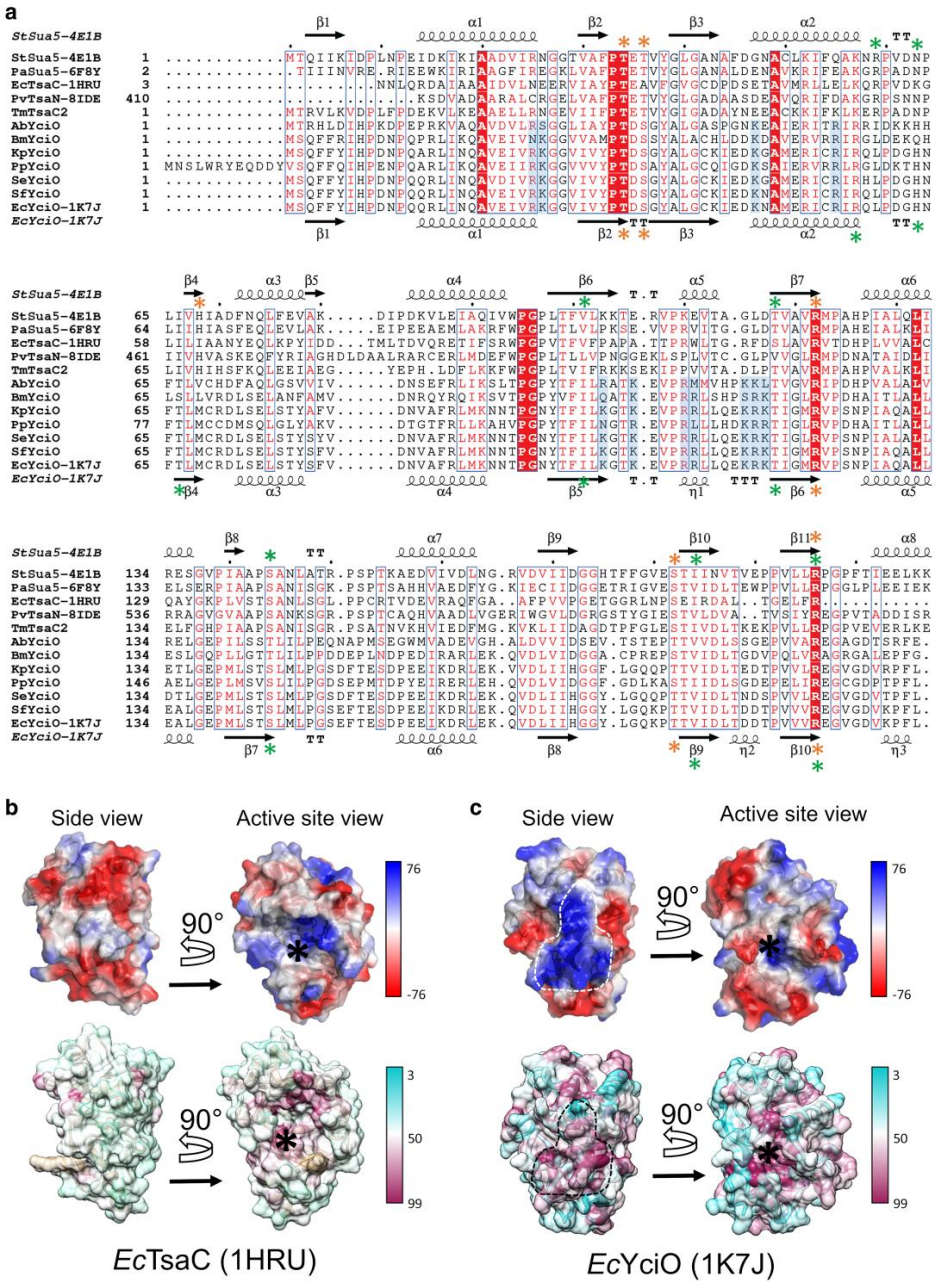
YciO harbors specific molecular surface features. a) Structure-based multisequence alignment of TsaC proteins, the TsaC domains of Sua5 proteins and YciO proteins, derived from available crystal structures and AlphaFold structural models. For crystal structures, the PDB IDs are indicated in the sequence name after the hyphen. Secondary structure elements from the crystal structures of *E. coli* TsaC and *E. coli* YciO are displayed above and below the sequences, respectively. YciO-specific conserved basic residues forming the positively charged surface patch of YciO are shaded in blue. Stars above and below the alignment indicate the crystallographically observed substrate binding residues in *St*Sua5 and the corresponding putative substrate binding residues in *Ec*YciO, respectively. Green stars indicate the Mg^2+^-ATP binding residues. Orange stars indicate the binding residues for the L-threonine substrate. *St*Sua5, *Sulfurisphaera tokodaii* Sua5 (UniProt ID Q9UYB2); *Pa*Sua5, *Pyrococcus abyssi* Sua5 (UniProt Q9UYB2); *Ec*TsaC, *Escherichia coli* TsaC (UniProt P45748); *Pv*TsaN, TsaC domain of *Pandoravirus* TsaN (UniProt A0A291ATS8). *Tm*TsaC2, *Thermotoga maritima* TsaC2 (by the authors, unpublished, UniProt Q9WZV6); *Ab*YciO, *Actinomycetales bacterium* YciO (UniProt A0A1R4F4R9); *Bm*YciO, *Burkholderia multivorans* YciO (UniProt A0A1B4MSV9); *Kp*YciO, *Klebsiella pneumoniae* YciO (UniProt A6T7X1); *Pp*YciO, *Pseudomonas putida* YciO (UniProt A5W0B1); *Se*YciO, *Salmonella enterica* TciO (UniProt A0A601PQ14); *Sf*YciO, *Shigella flexneri* YciO (UniProt P0AFR6); *Ec*YciO, *Escherichia coli* YciO (UniProt P0AFR4). b and c) Surface representations of the crystal structures of *Ec*TsaC b) and *Ec*YciO c), color-coded by surface electrostatic potential (top) and by positional sequence conservation score calculated from 4264 TsaC/Sua5 sequences and 1955 YciO sequences (bottom). The color keys for both panels are shown on the right. The conserved, YciO-specific, positively charged surface patch (6% of total molecular surface area) is encircled with a dashed line. The active center of TsaC and the putative active center of YciO are marked with asterisks. The figure illustrates that although the active site is conserved in both protein families, the positively charged surface patch is present and conserved only in the YciO family.

YciO is a member of the same Pfam family (PF01300) as TsaC/Sua5, and DeepEC predicts YciO has the same function as TsaC/Sua5. TsaC and Sua5 (the latter known in bacteria as TsaC2) are 2 types of the well-characterized L-threonylcarbamoyladenylate synthase (EC 2.7.7.87) that catalyzes the first step in the synthesis of the universal tRNA modified nucleoside *N*-6-threonylcarbamoyladenosine or t^6^A (TsaC and Sua5 have a common catalytic domain and differ by the presence of an additional domain in Sua5) (Su et al. 2022; Pichard-Kostuch et al. 2023). The function of TsaC/Sua5 was first elucidated in 2009 (El Yacoubi et al. 2009). A structure-based multisequence alignment comparing YciO and TsaC/Sua5 shows that the active site residues of TsaC are largely conserved in YciO, suggesting similar catalytic activities (Fig. 6a). YciO catalyzed the synthesis of L-threonylcarbamoyladenylate from ATP, L-threonine, and bicarbonate, in vitro (Kim et al. 2023). However, the activity reported (0.14 nM/min TC-AMP production rate) for *E. coli* YciO is more than 4 orders of magnitude weaker than that of *E. coli* TsaC (2.8 μM/min) at the same enzyme concentration and similar reaction conditions (Swinehart et al. 2020), consistent with the possibility of a missing partner or a different biological substrate for YciO.

**Fig. 7.**
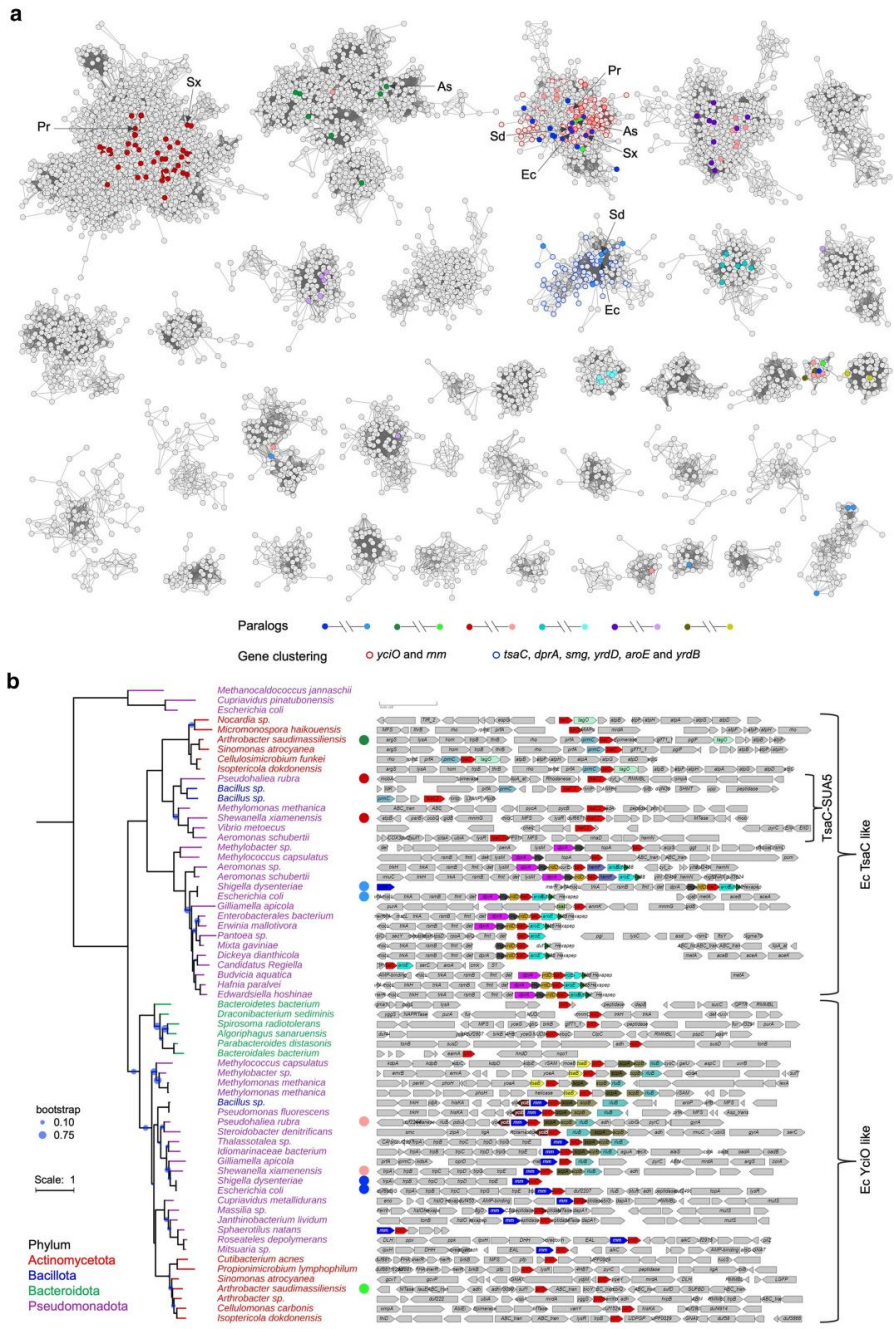
The genes encoding members of the *yciO* and *tsaC* have different genome neighborhood contexts. a) SSN of 54,820 PF01300 family members between 150 and 350 aa in length generated using EFI-EST. Each node in the network represents 1 or multiple sequences that share no less than 70% identity. An edge, represented as a line, is drawn between 2 nodes with AS higher than 70 (similar in magnitude to the negative base-10 logarithm of a BLAST e-value of 1E-70). Paralogs were colored in pairs as indicated. Node borders were colored by gene neighborhood context, *yciO* and *rnm* cluster (red) and *tsaC*, *dprA*, *smg*, *yrdD*, *aroE*, and *yrdB* cluster (blue). For better visualization, clusters of less than 40 nodes and singular nodes were hidden. The proteins used in the SSN are available in Supplementary Table 3. b) Phylogenetic tree of TsaC and YciO proteins. Sixty-two PF01300 proteins were selected from each major cluster in the family SSN and 3 RibB proteins (UniProt Q60364, Q46TZ9, and P0A7J0) were used as the outgroup. Bootstrap values less than 0.75 were indicated by blue dots. Branches and leaves were colored by phyla. TsaC2 proteins that contain YciO/TsaC (PF01300) and Sua5 (PF03481) domains are enclosed in a bracket. The gene neighborhood information is available in Supplementary Table 4. Selected example organisms were indicated by arrows and a 2-letter code in the SSN. Each corresponding genome neighborhood schematic was indicated by a circle in the same color as the node. As, *Arthrobacter saudimassiliensis*; Ec, *Escherichia coli*; Pr, *Pseudohaliea rubra*; Sd, *Shigella dysenteriae*; Sx, *Shewanella xiamenensis*.

YciO does not perform the same function as TsaC/Susa5 in vivo experiments (El Yacoubi et al. 2009; Gerdes et al. 2011). Genome neighborhood and structural data suggest that the function of YciO may be related to rRNA rather than tRNA metabolism. The evidence related to rRNA is as follows: (i) the structure of YciO exhibits a large positively charged surface predicted to interact with RNA (Jia et al. 2002) (Fig. 6b and c). This large positively charged surface is conserved in YciO proteins and is absent in TsaC proteins. (ii) In many species, *yciO* genes are colocalized with *rnm* genes (Fig. 7), which encode the recently characterized RNase AM, a 5′ to 3′ exonuclease that matures the 5′ end of all 3 ribosomal RNAs in *E. coli* (Jain 2020).

Models of enzyme evolution go through promiscuous stages (Ribeiro et al. 2023). TsaC is an enzyme predicted to have been present in the Last Universal Common Ancestor (Gagler et al. 2022; Pichard-Kostuch et al. 2023). YciO is a likely paralog of TsaC (Fig. 6), and as such, it is likely to have residual ancestral catalytic activity that can be detected in vitro. In summary, the functional puzzle is far from being solved for proteins of the YciO subgroup, and even if the existing data suggest a role in RNA metabolism, it cannot be the same as TsaC, and the EC number 2.7.7.87 prediction was given a CS of 0.

### Feature importance patterns identify limitations in the DeepEC model

**Fig. 8.**
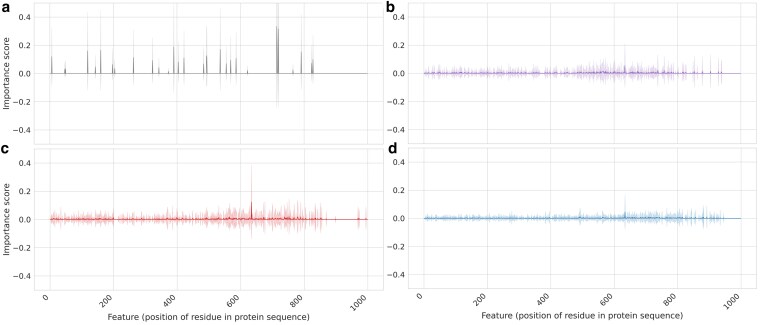
Residue-level feature importance profiles for DeepECTF highlight the distinct interpretability signatures associated with model predictions. Each line plot displays the normalized contribution (average feature importance computed via LIME) of individual residues across protein sequences for 4 categories: CORs a), paralog errors b), nonparalog errors c), and hallucinations or repetitive predictions d). Shaded regions represent standard deviation across instances within each category.

We explored if the use of XAI could help improve confidence in DeepECTF predictions using the 4 groups of proteins labeled with CORs, paralog errors, nonparalog errors, or REP errors (Fig. 4; Supplementary Table 1). Application of the XAI module to DeepECTF predictions enabled residue-level analysis of feature importance for EC prediction calls across protein sequences (Fig. 8). For CORs, the feature importance profiles identified a small number of residues—often corresponding to known catalytic or conserved domain positions, showing high contribution scores, indicating that features whose function relies on localized residues for function are more likely to be accurately predicted. In contrast, for all categories of erroneous predictions, feature importance profiles had small deviations from the null values, indicating that the model is not able to leverage information about the protein sequence in making its predictions.

## Discussion

Our expert curation of ML-based EC number predictions for *E. coli* “unknowns” proteins reveals that these methods still have a lot of room to improve. Biases, data imbalance (under- or overrepresented domains, motifs, and functions), generic EC numbers, lack of data structures that enable the inclusion of feature information, architectural limitations (e.g. incapacity of capturing complex patterns, inability to integrate diverse data sources, and lack of regularization), and poor uncertainty calibration all contribute to challenges in training computational models and are likely contributors in frequency-dependent predictions (Pucci et al. 2018; Urban et al. 2020; Mardikoraem and Woldring 2023; Shahbazi et al. 2023). Efforts to improve the quality and consistency of training data and avoid data leakage between training and testing data can help minimize errors, but metrics evaluating the uncertainty in predicted protein functions, or an assessment of the likelihood of the label assignment, need to become standard model outputs. In the absence of these metrics, XAI can be used to provide insights into feature importance and model behavior. Many advanced ML models, particularly deep neural networks such as DeepECTF, function as “black boxes,” where the prediction algorithm is difficult to interpret. XAI techniques can help to dissect “black-box” models and understand what input features are driving COR and incorrect prediction (Chennam et al. 2023) and provide insights about which data to include in future models to improve predictions.

Our study also shows that the model's lack of inclusion of the broader accumulated knowledge in the fields of protein structure and function, biochemical pathways, and the absence of a mechanism to identify “illogical” predictions led to erroneous predictions for most proteins of unknown functions, although the model was able to successfully propagate functions among proteins that were included in the training data. We also want to emphasize that in vitro activity alone is not sufficient to validate the function of a protein in vivo (García-Contreras et al. 2012; Punekar 2018). Indeed, enzymes evolve by duplication, divergence, and subsequent sub-, neo-, or hypofunctionalization (Ribeiro et al. 2023; Birchler 2025). In vitro activities of many enzymes can show promiscuity, which is useful for biotechnological applications (Robinson et al. 2020) but does not guarantee that the protein plays that specific role in vivo (Copley 2015) as shown here with the YciO example. Best practices in functional annotations of enzymes couple biochemical and contextual evidence and reflect the GO Consortium definitions, capturing the cellular component as well as the molecular and biological functions (Ashburner et al. 2000).

PLM-driven approaches are becoming mainstream tools for propagating known functional annotations among isofunctional proteins (https://www.uniprot.org/help/ProtNLM). Computational models will be further enabled by the integration of complementary evidence such as structural data to identify active site signature residues (Derry et al. 2025; Sajid et al. 2024; Yu et al. 2023), gene neighborhood context (Urhan et al. 2024; Hwang et al. 2024; Jha et al 2025), and chemical reaction specificity (Qian et al. 2024), all of which help to distinguish nonisofunctional paralogous subgroups (Ribeiro et al. 2023). Recent models like MAPred (Rong et al. 2024) align with modern goals of interpretability and transparency in ML models (Chennam et al. 2023; Rong et al. 2024; Derry et al. 2025) and implement a feature-dropping approach that represents a step toward a more quantitative and nuanced “self” evaluation of model predictions. The future in this space is exciting, even though the limits of these models are just now beginning to be understood (Muir et al 2025).

## Data Availability

Supplemental files are available at Figshare (https://doi.org/10.6084/m9.figshare.29602418.v2). Supplementary Table 1 contains the manual review of EC number predictions for 450 *E. coli* unknowns made by DEEP EC. Supplementary Table 2 contains gene neighborhood information for selected *yjdM* (IPR004624 family) genes shown in Fig. 5. Supplementary Table 3 includes the identifiers for the PF01300 family members used in Fig. 7 and SSN, and Supplementary Table 4 contains the gene neighborhood information for selected PF01300 family genes. Supplementary Data 1 contains the TsaC sequences used to generate positional conservation scores for Fig. 6. Supplementary Data 2 contains YciO sequences used to generate the positional conservation scores for Fig. 7.
